# WO_3_/BiVO_4_: impact of charge separation at the timescale of water oxidation†
†Electronic supplementary information (ESI) available: Including XRD, SEM, UV-visible absorption, current–voltage curves, IPCEs, additional TAS and TPC data, and EIS. See DOI: 10.1039/c8sc04679d


**DOI:** 10.1039/c8sc04679d

**Published:** 2019-01-16

**Authors:** Shababa Selim, Laia Francàs, Miguel García-Tecedor, Sacha Corby, Chris Blackman, Sixto Gimenez, James R. Durrant, Andreas Kafizas

**Affiliations:** a Imperial College London , Department of Chemistry , South Kensington , London , SW7 2AZ , UK . Email: a.kafizas@imperial.ac.uk; b Institute of Advanced Materials (INAM) , Universitat Jaume I , 12006 , Castelló de la Plana , Spain; c University College London , Department of Chemistry , Gordon Street , London , WC1H 0AJ , UK; d The Grantham Institute , Imperial College London , South Kensington , London , SW7 2AZ , UK

## Abstract

The four hole oxidation of water has long been considered the kinetic bottleneck for overall solar-driven water splitting, and thus requires the formation of long-lived photogenerated holes to overcome this kinetic barrier. However, photogenerated charges are prone to recombination unless they can be spatially separated. This can be achieved by coupling materials with staggered conduction and valence band positions, providing a thermodynamic driving force for charge separation. This has most aptly been demonstrated in the WO_3_/BiVO_4_ junction, in which quantum efficiencies for the water oxidation reaction can approach near unity. However, the charge carrier dynamics in this system remain elusive over timescales relevant to water oxidation (μs–s). In this work, the effect of charge separation on carrier lifetime, and the voltage dependence of this process, is probed using transient absorption spectroscopy and transient photocurrent measurements, revealing sub-μs electron transfer from BiVO_4_ to WO_3_. The interface formed between BiVO_4_ and WO_3_ is shown to overcome the “dead-layer effect” encountered in BiVO_4_ alone. Moreover, our study sheds light on the role of the WO_3_/BiVO_4_ junction in enhancing the efficiency of the water oxidation reaction, where charge separation across the WO_3_/BiVO_4_ junction improves both the yield and lifetime of holes present in the BiVO_4_ layer over timescales relevant to water oxidation.

## Abstract 2

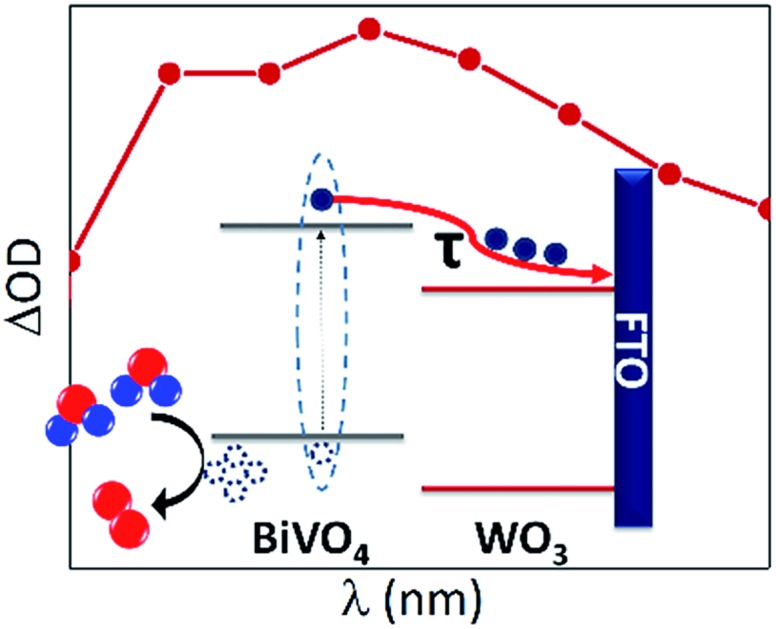
Unveiling the role of applied bias on the charge carrier dynamics in the WO_3_/BiVO_4_ junction during water oxidation.

## Introduction

The renewable generation of fuel is essential for combatting growing energy demands and rising atmospheric CO_2_ levels. Inorganic semiconductor systems have shown the best efficiencies for converting sunlight into fuels – most prominently in the production of hydrogen fuel from solar water splitting. However, it is well understood that the oxidation of water is the kinetically limiting step in the overall reaction, therefore developing materials that oxidise water efficiently is essential for improving the efficiency of water splitting systems.

Metal oxides such as BiVO_4_, WO_3_, TiO_2_, and α-Fe_2_O_3_ are amongst the most studied semiconductors for the water oxidation reaction.^1^–^6^ They are well known for their stability and deep valence band positions, making them attractive materials for oxidising water. Time-resolved studies have shown that water oxidation on metal oxides is kinetically slow, and typically takes place over several hundred milliseconds.^7^–^9^ Moreover, under operational conditions, the rate limiting step has been shown to involve the concerted reaction of multiple holes (or oxidised equivalents);^2^,^10^ similar to the manganese calcium cubane cluster in PS II.^11^,^12^ As such, metal oxides require long-lived holes to oxidise water efficiently.^13^–^15^ Several strategies have been employed to increase hole lifetime in metal oxides, such as the use of surface co-catalysts and passivation layers that inhibit surface electron–hole recombination.^16^,^17^ However, one of the most promising strategies for extending charge carrier lifetime is to couple semiconductors with staggered band alignment, which promotes the spatial separation of charge. This has been shown to synergistically enhance the activity in a number of systems including anatase/rutile TiO_2_, Cu_2_O/TiO_2_ and WO_3_/BiVO_4_.^5^,^18^,^19^ Using transient optical spectroscopy techniques, in agreement with previous reports, we show the enhancement in the performance of the WO_3_/BiVO_4_ heterojunction to be a direct result of fast electron transfer from BiVO_4_ into WO_3_.^20^,^21^ This leads to an overall decrease in recombination at timescales consequential to photocatalysis.

BiVO_4_ is emerging as one of the most popularly studied materials for driving the water oxidation reaction. The most active phase (monoclinic scheelite) is an n-type semiconductor with a band gap of 2.5 eV (*i.e.* can absorb ∼10% of the solar spectrum) and a deep valence band (∼+2.5 V_RHE_) providing a large thermodynamic driving force for the water oxidation reaction.^4^,^22^ However, it is often argued that this material suffers from poor electron transport properties, resulting in high recombination losses.^4^,^23^ Tungsten trioxide (WO_3_) is another frequently studied material for water oxidation. It possesses a wider indirect bandgap of 2.7 eV (*i.e.* can absorb ∼7% of the solar spectrum), a deeper valence band (∼+3.2 V_RHE_) and can typically oxidise water at less anodic potentials.^24^ When held at the thermodynamic water oxidation potential (1.23 V_RHE_), intrinsic BiVO_4_ and WO_3_ photoanodes typically show photocurrents in the region of 1–2 mA cm^–2^ at 1 sun irradiance, significantly less than their theoretical limit (BiVO_4_ ∼ 7.5 mA cm^–2^; WO_3_ ∼ 5.3 mA cm^–2^).^24^ Nevertheless, when these two materials are coupled together to form a WO_3_/BiVO_4_ junction, substantially higher photocurrents have been observed, approaching the theoretical limit for this system.^19^

Although the WO_3_/BiVO_4_ junction has been shown to exhibit high water oxidation efficiencies, to the best of our knowledge, there have been no *in operando* studies of charge carrier behaviour that interrogate the system at the timescale of water oxidation. To date, all transient absorption studies have focussed on the ultra-fast timescales (fs–ns), when charge generation and trapping processes occur with a lack of insight into charge carrier dynamics under operational conditions (*i.e.* applied potential).^20^,^21^,^25^ As such, the precise role of this junction in inhibiting electron–hole recombination and promoting water oxidation remains elusive.

In this article, we investigate the charge carrier dynamics in the WO_3_/BiVO_4_ junction during water oxidation using complementary transient absorption spectroscopy (TAS) and transient photocurrent (TPC) measurements. Our study shows that pre-μs electron transfer from BiVO_4_ to WO_3_, results in a significant improvement in the yield of holes accumulated at the surface in the heterojunction, with respect to BiVO_4_ alone. Moreover, anodic bias is found to substantially improve this electron transfer process, reducing recombination losses. These results thus shed new light on the role of this junction in facilitating synergistic improvements in water oxidation activity.

## Results

BiVO_4_ and WO_3_ thin films were prepared using metal organic decomposition (MOD) and aerosol assisted chemical vapour deposition (AA-CVD), respectively. X-ray diffraction confirmed the formation of monoclinic scheelite BiVO_4_ and monoclinic γ-WO_3_ (Fig. S1†). The deposited materials were flat and dense, as confirmed by SEM imaging (Fig. S2†). The films were further characterised by UV-Visible absorption spectroscopy (Fig. S3†), with the band edge observed at ∼500 nm for BiVO_4_ and ∼375 nm for WO_3_.

### Photoelectrochemical performance

**Fig. 1:**
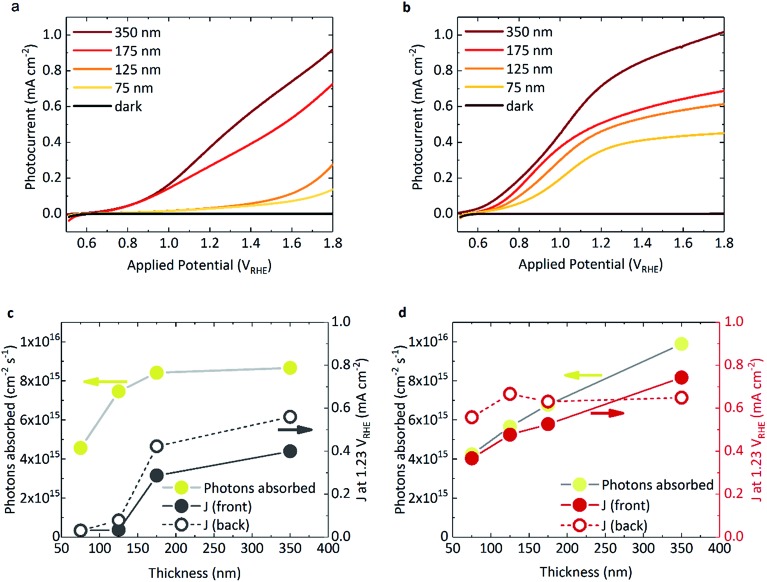
Photocurrent measurements of (a) BiVO_4_ and (b) WO_3_/BiVO_4_ films of varying BiVO_4_ thickness (and fixed WO_3_ thickness of 200 nm), under front illumination (*i.e.* through BiVO_4_ side) using simulated 1 sun irradiation, in 0.1 M phosphate buffer, pH 7. Photons absorbed by the sample compared with the photocurrent obtained at 1.23 V_RHE_ for front (solid circles) and back illumination (open circles) under simulated 1 sun irradiation for (c) BiVO_4_ and (d) WO_3_/BiVO_4_.

Fig. 1a and b shows the photocurrent response of BiVO_4_ and WO_3_/BiVO_4_, respectively, under 1 sun irradiance. The spectral irradiance for the simulated 1 sun light source is provided in Fig. S4.† The densely packed BiVO_4_ photoanodes studied herein display a strong performance dependence with film thickness (Fig. 1a). BiVO_4_ films that were ≤125 nm in thickness showed both a positively shifted onset potential and low photocurrents. However, at thicknesses of ≥175 nm, the onset potential shifted negatively (∼0.6 V_RHE_) with a concurrent substantial increase in photocurrent. The same trend was also observed with back irradiation (Fig. S5†). In contrast, when BiVO_4_ was deposited on WO_3_, much higher photocurrents were observed, even at low film thickness (Fig. 1b). The photocurrents obtained from the WO_3_ photoanode alone is negligible due to poor light harvesting properties and is presented in Fig. S5† for comparison. The absorbed photon to current efficiency (APCE) for the films under simulated 1 sun illumination are presented in Fig. S6b.† Interestingly, when comparing the absorbed photon density with the photocurrent obtained at 1.23 V_RHE_ as a function of BiVO_4_ film thickness, key differences between BiVO_4_ and WO_3_/BiVO_4_ were observed, as illustrated in Fig. 1c and d. Considering the behaviour of BiVO_4_ in Fig. 1c – back illumination generally gives rise to a better photocurrent response than front, attributed to poorer electron transport through the material with respect to hole transport.^23^ Although the photocurrent improves with increasing film thickness, it is clear that at lower thicknesses the performance is not limited by poor photon absorption (Fig. 1c). This trend with thickness is recognised as a signature characteristic of a “dead-layer effect” and has also been observed for hematite photoanodes (where the material becomes active once film thickness exceeds the inactive layer depth).^26^ Although the exact cause of the dead layer remains undefined, it is believed to arise from lattice mismatch at the semiconductor/FTO interface, which results in the formation of amorphous material close to the interface rich in trap states.^23^ The performance of BiVO_4_ has been shown to improve with the addition of a SnO_2_ underlayer,^23^,^27^,^28^ which may explain why the a WO_3_ underlayer in the WO_3_/BiVO_4_ system shows a substantial improvement in the activity of thin (≤125 nm) BiVO_4_ layers (Fig. 1a and b).

When illuminated from the front, the photocurrents observed in the composite WO_3_/BiVO_4_ films are largely governed by the light absorbed by the film, as shown in Fig. 1d. However, for both back and front illumination in Fig. S6b,† the APCE is greater at low thicknesses, which gradually decreases with increasing thickness of BiVO_4_. This is likely due to charge diffusion length limitations, where hole diffusion lengths in BiVO_4_ have been reported to be in the region of ∼100 nm.^29^ This effect is further illustrated when comparing the effect of back irradiation on APCE, which falls significantly from 85% to 40% when BiVO_4_ film thickness increases from 75 nm to 350 nm (Fig. S6b†). This is in stark contrast to bare BiVO_4_, and consistent with hole transport to the semiconductor–electrolyte surface being the limiting factor in WO_3_/BiVO_4_ (as opposed to electron transport to FTO and extraction from the material). A possible explanation for this observation could be related to the penetration depth of light and in turn, charge generation. When the films are illuminated from the back, charge generation is likely to be closer to the WO_3_/BiVO_4_ interface, as opposed to the BiVO_4_/electrolyte interface when illuminated from the front. Considering the former case, from the timescales determined from our transient absorption and transient photocurrent data (discussed later), we show that electron injection into WO_3_ from BiVO_4_ is much faster than electron injection into FTO.

### Transient absorption spectra

**Fig. 2:**
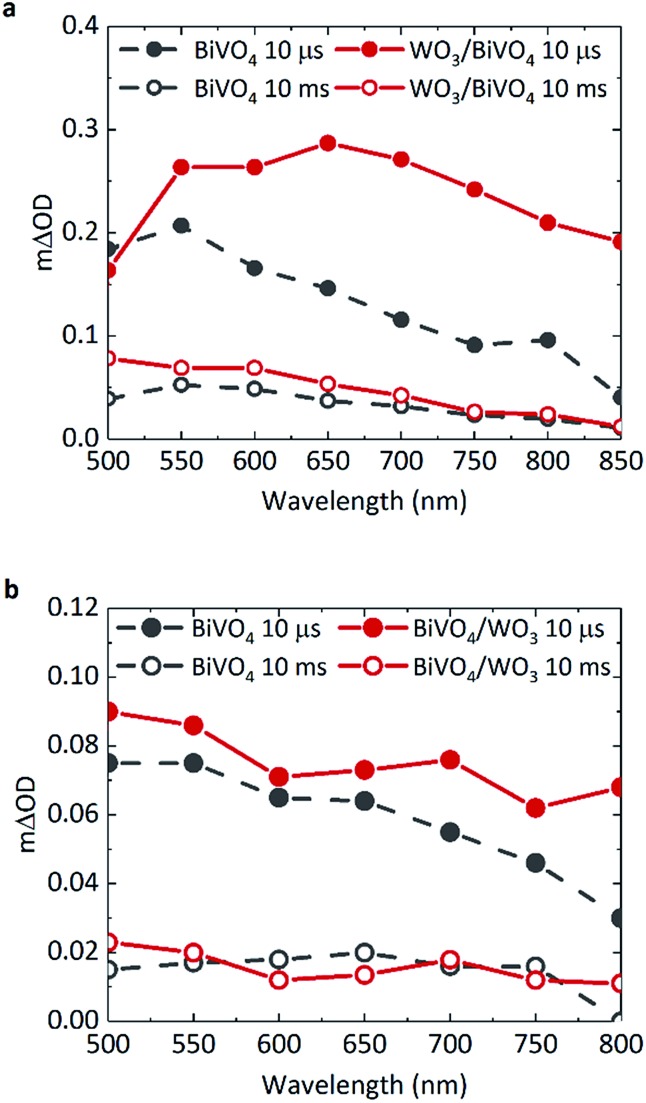
Transient absorption spectra of BiVO_4_ (grey) and WO_3_/BiVO_4_ (red) at 10 μs (solid) and 10 ms (open circles) under front illumination in 0.1 M phosphate buffer, with BiVO_4_ film thickness of 350 nm, pump fluence: 500 μJ cm^–2^. At (a) 1.23 V_RHE_ and (b) no applied bias (open circuit conditions).

TAS can be used to monitor time-resolved behaviour of photogenerated charge, therefore it can be employed to observe if (i) charge separation occurs across the WO_3_/BiVO_4_ interface and (ii) if this results in enhanced charge carrier lifetimes. Previous TAS studies of BiVO_4_ have shown that photogenerated holes in BiVO_4_ give rise to a broad absorption spectrum, absorbing most strongly at ∼550 nm.^8^ Due to the low absorption coefficient of electrons in BiVO_4_, the signal arising from these species was not detected or probed over the range of wavelengths studied herein. Fig. 2a shows the transient absorption spectra of BiVO_4_ and WO_3_/BiVO_4_ with applied anodic bias of 1.23 V_RHE_ at 10 μs and 10 ms. In order to isolate the effect of electron transfer from BiVO_4_ to WO_3_, films were excited with front illumination, *via* the BiVO_4_ side. This ensures that charge generation occurs predominantly within the BiVO_4_ layer, thus avoiding recombination pathways that occur when both electron and hole transfer occurs simultaneously at the interface by photoexciting both materials, as reported by Grigioni *et al.*^21^,^25^ According to the absorptance of each material (Fig. S3a†), 85% of the light at 355 nm is absorbed by the 350 nm thick BiVO_4_ layer, with 4% of the light likely to be absorbed by the 200 nm WO_3_ layer. Thus in our TAS studies, BiVO_4_ film thickness was held constant at 350 nm to maximise light absorption in the BiVO_4_ layer. The observed trends in charge carrier dynamics were verified using an excitation wavelength at which the WO_3_ layer is not excited (see Fig. S7c in ESI†).

The shape of the transient absorption spectrum of BiVO_4_ under anodic bias, presenting a maximum at 550 nm, is in agreement with previous reports (Fig. 2a, grey trace).^8^ However, the WO_3_/BiVO_4_ heterojunction shows a different spectral shape at early timescales, with an additional absorption feature at longer wavelengths that is not present in bare BiVO_4_ (Fig. 2a, red trace). This feature disappears by 10 ms, following which the spectrum of the heterojunction is analogous to that of bare BiVO_4_, but with a ∼two-fold increase in amplitude at 500 nm. Previous works have shown that photogenerated electrons in WO_3_ present a broad transient absorption feature at around 800 nm, which extends into the near-infrared region.^30^,^31^ Given we also observe this absorption feature in the heterojunction when the excitation wavelength does not excite WO_3_ (Fig. S7c, ESI†), this shows that at early timescales (microseconds) the heterojunction contains holes in BiVO_4_ and electrons in WO_3_. This indicates pre-μs electron transfer from BiVO_4_ to WO_3_; in accordance with previous reports.^20^ The electron signal in WO_3_ will be discussed in further detail later.

In Fig. 2b, there is no significant enhancement of carrier lifetime in the absence of any applied potential for the heterojunction in comparison to bare BiVO_4_. Additionally, in contrast to Fig. 2a, the signal contribution from electrons transferred to WO_3_ is less pronounced in the absence of applied potential. This lack of enhancement indicates the need for applied bias to increase the efficiency of charge transfer across the interface, and more importantly, shows that the conduction band offset alone is insufficient in driving electron transfer. Loiudice *et al.* has previously reported the transient absorption decay of holes in BiVO_4_ and the heterojunction in air, where a substantial enhancement of hole lifetime in the heterojunction is observed in the ms–s region, whilst the bare BiVO_4_ signal decays to zero by 10 ms.^32^ However in our study, measurements are conducted in electrolyte, which can induce band bending at the semiconductor/electrolyte interface that can prolong photogenerated hole lifetimes in bare BiVO_4_. In electrolyte, the dark open circuit potential obtained for bare BiVO_4_ is around ∼+0.6/0.7 V_RHE_, which when compared to the flat band potential of BiVO_4_ (∼+0.1 V_RHE_),^8^ signifies the presence of substantial band bending that can accommodate hole accumulation at the surface. Thus, in the TAS spectra presented in Fig. 2b, no significant enhancement in photogenerated carrier lifetime for the heterojunction system (red trace) is observed in the μs–s timescale, compared to bare BiVO_4_ (grey trace) under open circuit conditions.

### Transient absorption kinetics of surface holes

**Fig. 3:**
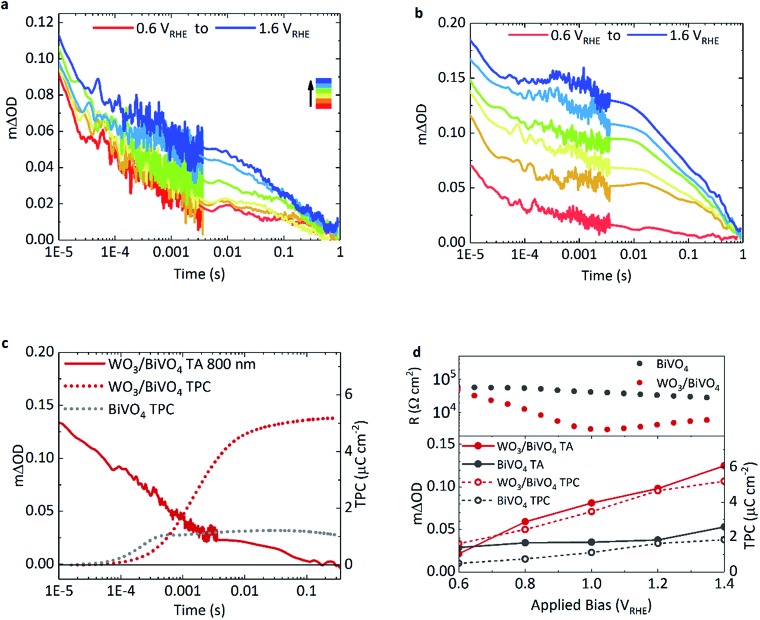
Transient absorption kinetics of BiVO_4_ and WO_3_/BiVO_4_ when excited at 355 nm under front illumination, with applied anodic bias (0.2 V_RHE_ interval) in 0.1 M phosphate buffer with BiVO_4_ film thickness of 350 nm, pump fluence: 500 μJ cm^–2^. TA decay traces of holes probed at 500 nm in (a) BiVO_4_ and (b) WO_3_/BiVO_4_. (c) TA trace of electrons in WO_3_/BiVO_4_ (red, solid line) at 1.23 V_RHE_ probed at 800 nm accompanied with the integrated transient photocurrent traces at the same bias for BiVO_4_ (grey, dotted) and WO_3_/BiVO_4_ (red, dotted). (d) The dependence of the film resistance as a function of applied bias (top panel) and the TA amplitude at 1 ms of holes at 500 nm with anodic bias (solid circles), in BiVO_4_ (grey) and WO_3_/BiVO_4_ (red) compared with the corresponding integrated transient photocurrent extracted from the films as a function of applied bias (open circles) (bottom panel).

The effect of applied bias on the charge carrier dynamics was monitored in BiVO_4_ and WO_3_/BiVO_4_. As mentioned earlier, the electron signal in WO_3_ is broad, stretching into the NIR, with a peak maximum at around 800 nm. Holes in BiVO_4_ were probed at 500 nm to minimise overlap with WO_3_ electron absorption. Fig. 3a and b show kinetic traces of the photogenerated holes for BiVO_4_ and WO_3_/BiVO_4_ respectively as a function of applied potential. While the lifetime and population of holes increase with increasing anodic bias in both systems, the increase is more substantial in the heterojunction (Fig. 3b) in comparison to bare BiVO_4_ (Fig. 3a). Anodic bias results in the formation of a wider space charge layer at the semiconductor/electrolyte interface, increasing band bending and facilitating greater hole accumulation at the surface. In contrast, in the presence of a hole scavenger, shown in Fig. S9,† hole accumulation in the heterojunction is much less bias dependent, due to a more facile oxidation of sulphite in comparison to water. The biphasic nature of the hole decay dynamics observed in bare BiVO_4_, consistent with previous reports, consists of trap-mediated bimolecular recombination that dominates at earlier timescales (<μs–ms) and water oxidation coupled with back electron–hole recombination processes that take place over slower timescales (ms–s).^8^,^9^,^33^,^34^ Fittings of the kinetic traces in Fig. 3a and b are provided in the ESI (Fig. S11†).

Back electron–hole recombination is a process whereby photogenerated holes, which accumulate at the surface of the semiconductor owing to band bending, recombine with electrons in the bulk due to coulombic attraction (this is sometimes referred to as surface recombination), thus resulting in a backflow of electrons from the external circuit into the photoanode. It has been extensively studied in α-Fe_2_O_3_, BiVO_4_ and TiO_2_.^35^–^37^ In bare BiVO_4_, at early timescales, bimolecular recombination, even with appreciable anodic bias, plays a significant role. However, this recombination pathway is significantly suppressed in the WO_3_/BiVO_4_ heterojunction with increasing anodic bias, resulting in more substantial increase in hole lifetimes in BiVO_4_ (Fig. 3b). The requirement for long-lived holes is a consequence of the slow kinetics of water oxidation on BiVO_4_ (and metal oxides in general), which is on the order of 0.7–1 s^–1^ on BiVO_4_ under pulsed laser illumination.^37^ These kinetics are apparent as the slow decay phase from 0.01 ms to 1 s in Fig. 3a and b. No acceleration of this slow decay phase, assigned to water oxidation, is observed for the heterojunction over bare BiVO_4_, as the rate of catalysis is limited by the material in which holes accumulate. On the other hand, in the heterojunction, a substantial improvement in charge carrier separation results in an increase in the population of long lived holes, and an overall increase in water splitting activity.

### Electron transfer, charge extraction and performance

Transient photocurrent measurements monitor the charge extraction processes from the photoanode following photoexcitation, and therefore allow direct probing of the timescale of charge injection into the back contact.

Consequently, we can directly compare the transient optical signal of electrons against the timescale of charge extraction. This is shown in Fig. 3c where the transient absorption decay at 800 nm (red, solid), probing primarily WO_3_ electrons, is plotted against the integrated transient photocurrent (charge extraction) from the photoanode (red, dotted), at 1.23 V_RHE_.

Firstly drawing our focus on the TPC data in Fig. 3c, we observe a 5-fold increase in the integrated transient photocurrent (charge) extracted from the heterojunction (red, dotted) compared to bare BiVO_4_ (grey, dotted), a direct consequence of effective charge separation suppressing bulk recombination in the heterojunction. We also observe that the onset of charge extraction is around 50 μs for the heterojunction, which is an order of magnitude later than the bare BiVO_4_ photoanode. This slower extraction from the heterojunction is due to the slower electron transport properties of WO_3_ compared with BiVO_4_. It has been reported that electron transport in WO_3_ is the slowest amongst common metal oxides (*i.e.* BiVO_4_, TiO_2_ and Fe_2_O_3_).^31^ A direct comparison of charge extraction times in the different systems studied herein is shown in Fig. S8e,† which confirms electron extraction is indeed slower for WO_3_ as a stand-alone material than it is for BiVO_4_. This is consistent with the previous report, and manifests as an overall slower charge extraction from the heterojunction system.

Secondly, in the heterojunction, comparing the optical decay of electrons (red, solid) with charge extraction (red, dotted) in Fig. 3c, we find that the optical signal decays prior to the onset of charge extraction (<50 μs). This indicates that a portion of electrons are lost at early timescales and not collected (<50 μs, ∼30% loss of signal). In addition, from the photogenerated hole kinetics of the heterojunction in Fig. 3b, we also observe a similar decay of the signal at early timescales (<50 μs), which we tentatively attribute to the bimolecular recombination of electrons and holes at the WO_3_/BiVO_4_ interface before charge extraction takes place. We find that most of the charge is extracted by 10 ms in the heterojunction (Fig. 3c, red dotted). We also monitor the electron extraction from the photoanode optically in the transient absorption spectra in Fig. 2a (red, open circle), where the spectra of the heterojunction at 10 ms (once electron extraction is complete) resembles that of holes accumulated at the surface of BiVO_4_ (grey, open circles). The bias dependence of the optical signal arising from electrons transferred to WO_3_ is presented in Fig. S8a,† which shows how charge separation across the WO_3_/BiVO_4_ interface improves with anodic potential.

Fig. 3d compares the relationship between the optical signal of holes in BiVO_4_ at 1 ms with the total charge extracted from the photoanodes and the overall resistance of the photoanodes, as a function of applied potential. If we also compare the optical signal of electrons transferred to WO_3_ prior to the onset of charge extraction against the charge extracted, we see a similar relationship (Fig. S8f†). Firstly, turning our attention to the transient absorption and photocurrent data, we observe a direct correlation between extended hole carrier lifetime at 1 ms and increased charge extraction, from moderate to high anodic bias. Moreover, compared to bare BiVO_4_, a greater proportion of the charge separated at 1 ms in the heterojunction manifests as charge that can be extracted. From the plot, it is evident that charge separation in the heterojunction improves with increasing applied potential compared to BiVO_4_, which is in good agreement with previous electrochemical impedance spectroscopy (EIS) studies.^38^ This further correlates with film resistance determined from impedance measurements herein, where the resistance of the heterojunction lowers considerably with increasing bias in relation to bare BiVO_4_. In comparison, this resistance, originating from the slope of the *j*–*V* curve, is almost constant for the pristine BiVO_4_ sample for the voltage range examined.

## Discussion

Our results show that the WO_3_/BiVO_4_ heterojunction exhibits a higher water oxidation performance than bare BiVO_4_ due to faster electron transfer from BiVO_4_ to WO_3_ than BiVO_4_ to FTO. This results in more effective charge separation that limits bimolecular recombination losses. This is consistent with the resistance calculated for the heterojunction being lower than that of the bare materials, leading to lower bulk recombination losses.^38^ In bare BiVO_4_, we observe from TPC measurements that charge injection into FTO begins from around 20 μs but the majority of the charge is extracted after 100 μs (Fig. 3c). However, in the heterojunction, our TAS measurements (Fig. 2a) show that electrons were transferred from BiVO_4_ to WO_3_ on the pre-μs timescale. This fast electron transfer to WO_3_ minimises the recombination loss pathways that can occur when the electrons and holes reside within the same material. Prolonging charge lifetime through charge transfer and vectoral separation has evolved in nature over billions of years and is specifically employed in photosystem II (PSII) for driving water oxidation.^39^,^40^ Taking inspiration from PSII, where an assortment of processes such as light absorption, charge separation and transfer occur successively, we can mimic this approach to maximise the efficiency of photoelectrodes for water splitting.^41^ By forming a staggered heterojunction like WO_3_/BiVO_4_, we demonstrate how this system spatially separates photogenerated charge, and thus prolongs the lifetime and population of photogenerated holes that drive the kinetically limiting water oxidation reaction.

**Scheme 1:**
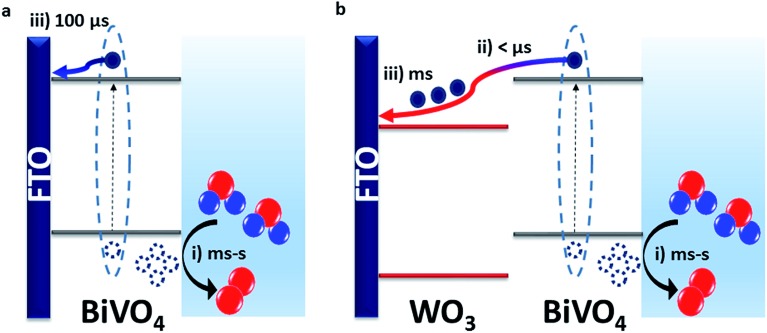
Schematic representation of charge transfer processes in (a) BiVO_4_ and (b) WO_3_/BiVO_4_; where (i) denotes holes reacting with water to form oxygen, (ii) is the electron transfer from BiVO_4_ to WO_3_ and (iii) is the electron transfer from the semiconductor to the FTO.

Fast electron transfer from BiVO_4_ to WO_3_ highlights that, in the bare BiVO_4_ films, slower electron injection into FTO limits the performance. This slower injection into FTO combined with the dead layer effect observed for thin bare BiVO_4_ films underlines the limitations of the FTO/BiVO_4_ contact, and why it has been shown to improve in presence of underlayers that form a more favourable interface.^23^ We can directly compare the timescales of charge extraction in the WO_3_/BiVO_4_ heterojunction with that of the bare WO_3_ and BiVO_4_ in Fig. S8e.† From this, we can draw two main conclusions: (i) charge extraction at these studied thicknesses, is fastest in BiVO_4_ and follows the order: BiVO_4_ < WO_3_ < WO_3_/BiVO_4_. This explains the order of magnitude difference in charge extraction timescales between BiVO_4_ and WO_3_/BiVO_4_, owing to slower charge transport through the WO_3_ layer. (ii) Although electron extraction into the back contact is slower in WO_3_, this doesn't limit the overall performance of the heterojunction as it does for bare BiVO_4_, because the electrons and holes reside in separate materials, and this spatial separation of charge inhibits the bulk recombination. This is in agreement with the findings of a previous impedance spectroscopy based study of mesoporous WO_3_/BiVO_4_ heterostructures.^38^ Using time-resolved optical spectroscopy techniques we were able to directly observe the electrons that were transferred from BiVO_4_ to WO_3_, and conclude that the performance enhancement in the heterojunction is a direct result of faster electron injection into WO_3_ from BiVO_4_ than injection into FTO directly from BiVO_4_. These charge transfer are illustrated in Scheme 1.

We note that while photocurrents under simulated 1 sun irradiation (Fig. 1a and b) demonstrate a ∼2-fold increase in photocurrent in the heterojunction compared with bare BiVO_4_ at 1.23 V_RHE_, our transient photocurrent measurements show an ∼5-fold increase in the photocharge extracted following excitation at 355 nm. This difference in performance can be attributed to two main factors: (i) the wavelength dependence of sample performance and (ii) the differing photoresponse of our samples to pulsed and continuous light sources. Firstly, considering (i), the wavelength dependence of the photoresponse is reflected in the IPCE of the films shown in Fig. S6a.† The enhancement in efficiency of the heterojunction with respect to bare BiVO_4_ is more pronounced at 355 nm compared to longer wavelengths. Therefore, when irradiated with a continuous Xe-lamp light source at simulated 1 sun, as seen in Fig. S4,† most of the photon flux of the light source lies in the region where the enhancement manifests as a two-fold increase in performance. Secondly considering (ii), the photoresponse of a material can vary when illuminated with an excitation pulse or a continuous light source. This indicates that the WO_3_/BiVO_4_ heterojunction is more resilient to bimolecular recombination than BiVO_4_ alone.

Hole lifetimes are found to improve with increasing anodic bias where the initial TA signal amplitude of the heterojunction is heavily dependent upon the bias applied. At potentials close to the photocurrent onset (0.6 V_RHE_), the amplitude for holes in the heterojunction at 10 μs is lower than it is for bare BiVO_4_. This may be a result of recombination processes at the junction interface being predominant on the pre-μs timescale, which can be circumvented by increasing the anodic bias applied (and hence the band bending of BiVO_4_ at the semiconductor/liquid interface) to favour charge separation. Although pre-μs carrier dynamics are not probed in this study, there have been other TAS studies of the charge carrier dynamics on the ultra-fast timescale for this heterojunction in the absence of applied bias.^21^,^25^,^32^ Their findings suggest the presence of loss mechanisms due to recombination across the interface of the materials following fast charge transfer to WO_3_.

Previous studies have shown that the typical conduction band positions of WO_3_ and BiVO_4_ are +0.41 V_RHE_ and +0.02 V_RHE_ whilst the valence band positions are expected to be around +3.18 V_RHE_ and +2.53 V_RHE_ for WO_3_ and BiVO_4_, respectively, forming a type II staggered heterojunction (Scheme 1b).^42^ Hence, there is a thermodynamic driving force for electrons generated in the conduction band of BiVO_4_ to transfer to the conduction band of WO_3_. However, our bias dependence studies show that at low to modest bias (≤0.6 V_RHE_), electron transfer to WO_3_ is inefficient, and any spatial separation of charge does not prevent interfacial recombination. Modest applied potentials (>0.6 V_RHE_) are required for charge separation to occur efficiently. In accordance with typical band alignments drawn for n–n heterojunctions where bands are assumed to be pinned at the interface, Fermi level alignment would give rise to formation of space charge layers at the interface that can hinder charge transfer (*i.e.* a Schottky barrier).^43^–^45^ Therefore, there is scope for finding other combinations of semiconductors with staggered band alignment that can form a more favourable interface that precludes the need for applied bias to enhance charge separation. Furthermore, this should result in an earlier onset of photocurrent. Further studies of the exact nature of the alignment at the interface can shed light into the need for anodic bias to facilitate efficient charge separation in this system, but this is beyond the scope of this study.

Overall, we find that the n–n type heterojunction increases photocurrent by minimising bimolecular recombination, specifically at timescales that directly compete with water oxidation. As electrons are transferred to the WO_3_ on the pre-μs timescale, this significantly reduces the proportion of charge that undergo bimolecular recombination compared to bare BiVO_4_ for which charge injection into FTO lies on the ∼20–100 μs timescale. On the other hand, slow charge transport properties of WO_3_ can give rise to interfacial recombination observed prior to charge extraction from the heterojunction photoanodes, leaving scope for other materials to serve as better electron acceptor layers.

## Conclusions

For the first time, to our knowledge, we have measured the time-resolved behaviour of charge carriers in WO_3_/BiVO_4_ heterojunction photoanodes *in operando* at timescales relevant to water oxidation. WO_3_/BiVO_4_ junctions exhibit superior performance with respect to the bare materials. Our findings suggest that the performance enhancement results from a combination of factors. These include reduced electron–hole recombination processes resulting from better charge transfer from BiVO_4_ to WO_3_ than direct injection into FTO. Moreover, the presence of a WO_3_ underlayer eradicates the dead-layer effect observed in very thin films of BiVO_4_ (≤125 nm), significantly improving photoelectrochemical performance. Our transient absorption studies show that the band alignment present in the WO_3_/BiVO_4_ heterojunction does not enhance charge lifetime alone, and requires anodic bias (>0.6 V_RHE_) to increase the lifetime and population of holes that oxidise water on the ms–s timescale. This work demonstrates an effective example of how inorganic systems can be used to increase charge carrier lifetime, similar to natural photosynthetic systems, and compete with the slow kinetics of water oxidation.

## Experimental

### Materials fabrication

All materials were prepared on FTO substrate (2.5 cm × 2.5 cm, TEC 15, Hartford Glass Co.). Before deposition, the substrates were washed with a standard glass cleaner, followed with deionised water, acetone and iso-propanol and subsequently heated to 500 °C for 10 minutes. All chemicals were purchased from Sigma-Aldrich unless specified otherwise.

WO_3_ films were prepared using aerosol assisted chemical vapour deposition (AA-CVD) method previously reported.^46^,^47^ In brief, the substrate is heated to 325 °C. A stock solution of W(CO)_6_ (0.6 g, 11.4 mM) was prepared in a 1 : 2 mixture of methanol and acetone. Depositions of 1 mL of the stock solution results in a film thickness of ∼200 nm, as verified previously.^46^ The solution was aerosolised with an ultrasonic humidifier (2 MHz, Liquifog, Johnson Matthey), before being carried into the reaction chamber with N_2_ as the carrier gas (0.5 mL min^–1^). Following complete transfer of precursor solution, the substrate is cooled to room temperature over N_2_ flow, forming oxygen deficient WO_3–*x*_ which requires further annealing at 500 °C in air for 1 hour to yield WO_3_.

BiVO_4_ films were prepared using a modified metal organic decomposition, previously reported.^8^,^48^ Bismuth nitrate pentahydrate (0.1455 g, 200 mM) was dissolved in acetic acid (1.5 mL, VWR) and vanadyl acetyl acetone (0.0768 g, 30 mM) was dissolved in acetyl acetone (10 mL, VWR). The two solutions were then mixed and stirred at room temperature for 30 minutes to prepare sol–gel. The sol–gel mixture was subsequently deposited by spin-coating. 50 μL of the solution was used per layer. Following the deposition of each layer, the substrates were calcined to 450 °C for 10 minutes. This process was repeated accordingly for the desired film thicknesses. After the deposition of the final layer, the films were calcined at 450 °C for 5 hours, forming densely packed BiVO_4_ thin films. For the purposes of this study, films of 3, 5, 7 and 14 layers were prepared, giving thicknesses of approximately 75 nm, 125 nm, 175 nm and 350 nm, respectively. For the heterojunction films, BiVO_4_ was deposited on the as prepared WO_3_ films.

### Materials characterisation

UV-visible spectroscopy was measured using a Shimadzu UV-2700 equipped with an integrated sphere. XRD patterns were measured using a modified Bruker-Axs D8 diffractometer with parallel beam optics equipped with a PSD LinxEye silicon strip detector. The instrument uses a Cu source for X-ray generation (*V* = 40 kV, *I* = 30 mA) with Cu K_α1_ (*λ* = 1.54056 Å) and Cu K_α2_ radiation (*λ* = 1.54439 Å) emitted with an intensity ratio of 2 : 1. The incident beam was kept at 1° and the angular range of the patterns collected between 10 ≤ 2*θ*° ≤ 66 with a step size of 0.05°. SEM images were obtained with a LEO 1525 scanning electron microscope (FESEM, 5 kV).

### Photoelectrochemical characterisation

Photoelectrochemical characterisation was carried out in a three-electrode cell, with our photoanode material placed at the working electrode, a Pt mesh at the counter electrode and a saturated KCl_aq_ Ag/AgCl as the reference electrode (Metrohm). All measurements were carried out in 0.1 M phosphate buffer_(aq)_ (pH 7) for BiVO_4_ and WO_3_/BiVO_4_ films, and 0.1 M H_2_SO_4(aq)_ (pH 1) for WO_3_ films. Unless specified otherwise, TAS, TPC and PEC were measured *via* front side (electrode–electrolyte) irradiation. Applied potentials measured *vs.* Ag/AgCl (*V*_Ag/AgCl_) were converted to applied potentials *vs.* the reversible hydrogen electrode (*V*_RHE_) using the Nernst equation:

1 *V*_RHE_ = *V*_Ag/AgCl_ + 0.0591 × pH + *V*0Ag/AgCl

where *V*0Ag/AgCl is the standard potential of the Ag/AgCl reference electrode in sat. KCl (197 mV).

The impedance spectroscopy (IS) measurements were carried out in a potentiostat/galvanostat Autolab (model PGSTAT-30). Measurements were performed in a three-electrode configuration, where a platinum wire was used as counter electrode, the sample under study was used as the working electrode, and an Ag/AgCl (3 M KCl) electrode was used as the reference electrode. An aqueous phosphate buffer solution at pH 7 (0.1 M NaH_2_PO_4_/Na_2_HPO_4_) was used as electrolyte. The electrochemical measurements were referred to the reversible hydrogen electrode (RHE) through the Nernst equation (eqn (1)), where *V*0Ag/AgCl (3 M KCl) is 0.210 V. Impedance measurements under illumination conditions were carried out with a 300 W Xe lamp using a thermopile to adjust light intensity at 10 mW cm^–2^ (0.1 sun). The resistance was calculated using a model reported previously.^38^

### TAS and TPC measurements

Transient absorption spectroscopy (TAS) measurements were carried out on a home-built configuration consisting of a Nd:YAG laser (OPOTEK Inc., Opolette 355 laser system, 7 ns pulse width) at 355 nm (and 450 nm for selective excitation of BiVO_4_). The laser frequency was set to 0.7 Hz, and the laser intensity adjusted to 500 μJ cm^–2^ (and 400 μJ cm^–2^ for 450 nm excitation). The light source used for the probe beam is a 100 W Bentham IL1 tungsten lamp coupled to a monochromator (OBB-2001, Photon Technology International). To filter scattered laser light, the probe beam passed through longpass filters and another monochromator set to the same wavelength as the probe beam. To detect the transmitted photons, a Si-photodiode was utilised (Hamamatsu). The obtained data were processed through an amplifier (Costronics) and subsequently recorded by an oscilloscope (Tektronics TDS 2012B) for the μs–ms timescale, and the ms–s timescale data were recorded with a DAQ card (National Instruments, NI USB-6211). For each transient absorption decay, the data were averaged over 100 laser pulses. The system and data acquisition were controlled by a home programmed Labview software. The electrical bias was applied using an Autolab potentiostat (PGSTAT 101, Metrohm). Transient photocurrent (TPC) data were obtained using a modified TAS setup where the decays of the transient current signal were recorded on the scope coupled to a ministat (Sycopel Scientific Ltd.).

## Conflicts of interest

There are no conflicts to declare.

## Supplementary Material

Supplementary information
