# Supplementary material for: WO3/BiVO4: impact of charge separation at the timescale of water oxidation
Source: Chem Sci. 2019 Jan 16;10(9):2643–52. doi: 10.1039/c8sc04679d (PMC6419945; doi:10.1039/c8sc04679d)
Supplement: Supplementary file 1 [file SC-010-C8SC04679D-s001.pdf]

Electronic Supplementary Material (ESI) for Chemical Science.
This journal is © The Royal Society of Chemistry 2019

$WO_3/BiVO_4$: Impact of charge separation at the timescale of water oxidation – Supplementary Information

Shababa Selim,[a] Laia Francàs,[a] Miguel García-Tecedor,[b] Sacha Corby,[a] Chris Blackman,[c] Sixto Gimenez,[b] James R. Durrant,[a] Andreas Kafizas[a,d*]

[a]Imperial College London, Department of Chemistry, South Kensington Campus, London, SW7 2AZ, UK.

[b]Institute of Advanced Materials (INAM), Universitat Jaume I, 12006, Castelló de la Plana, Spain.

[c]University College London, Department of Chemistry, Gordon Street, London, WC1H 0AJ, UK.

[d]The Grantham Institute, Imperial College London, South Kensington Campus, London, SW7 2AZ, UK.

*corresponding author: a.kafizas@imperial.ac.uk

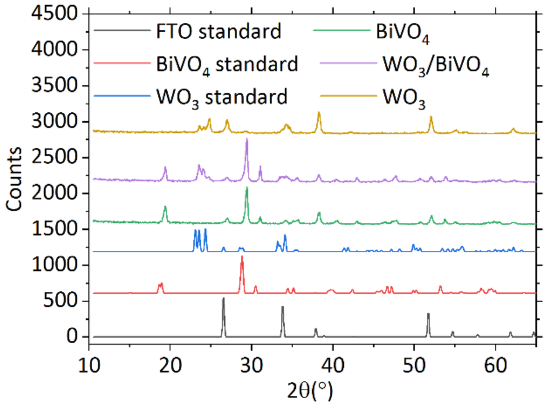

Fig. S1 Typical XRD patterns of $BiVO_4$, WO_3 and $WO_3/BiVO_4$ shown alongside reference standards (monoclinic scheelite $BiVO_4$, monoclinic WO_3 and tetragonal cassiterite F: SnO_2).

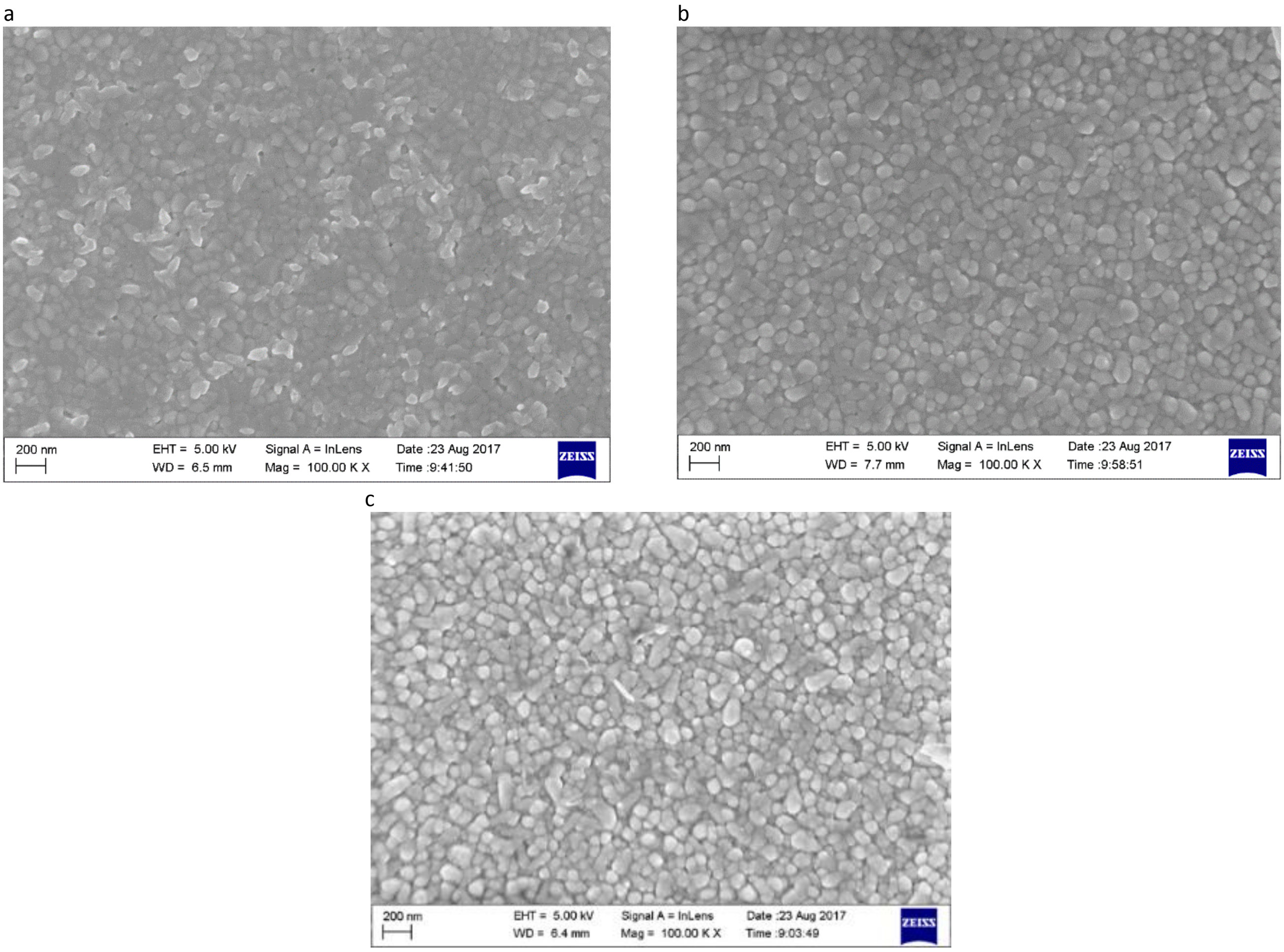

Fig. S2 SEM images of (a) $BiVO_4$, (b) WO_3 and (c) $WO_3/BiVO_4$. Scale bar represents 200 nm.

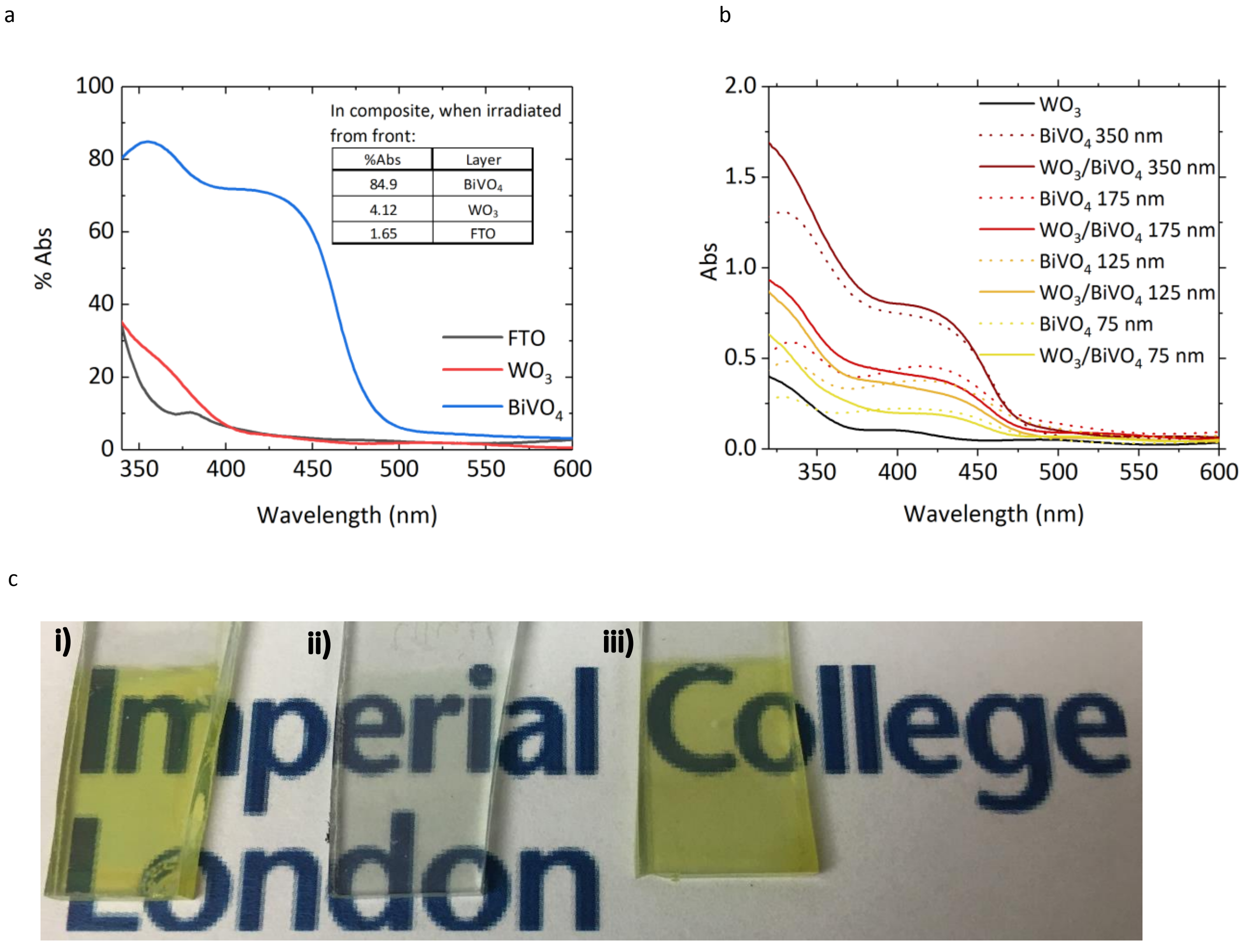

Fig. S3 UV-Vis spectra of photoanode films measured using a Shimadzu UV-2700 equipped with an integrated sphere. (a) Absorptance of films taking into account of transmittance, diffuse reflectance and specular reflectance. (b) UV-Vis spectra of bare $BiVO_4$ and $WO_3/BiVO_4$ with varying thicknesses of $BiVO_4$. (c) images of the films: (i) $BiVO_4$, (ii) WO_3 and (iii) $WO_3/BiVO_4$

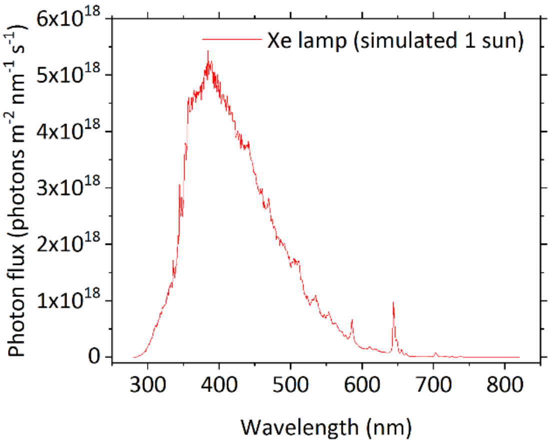

Fig. S4 Spectral output of the Xe lamp at 100 mW.cm^{-2} with a KG3 filter (simulated 1 sun irradiance).

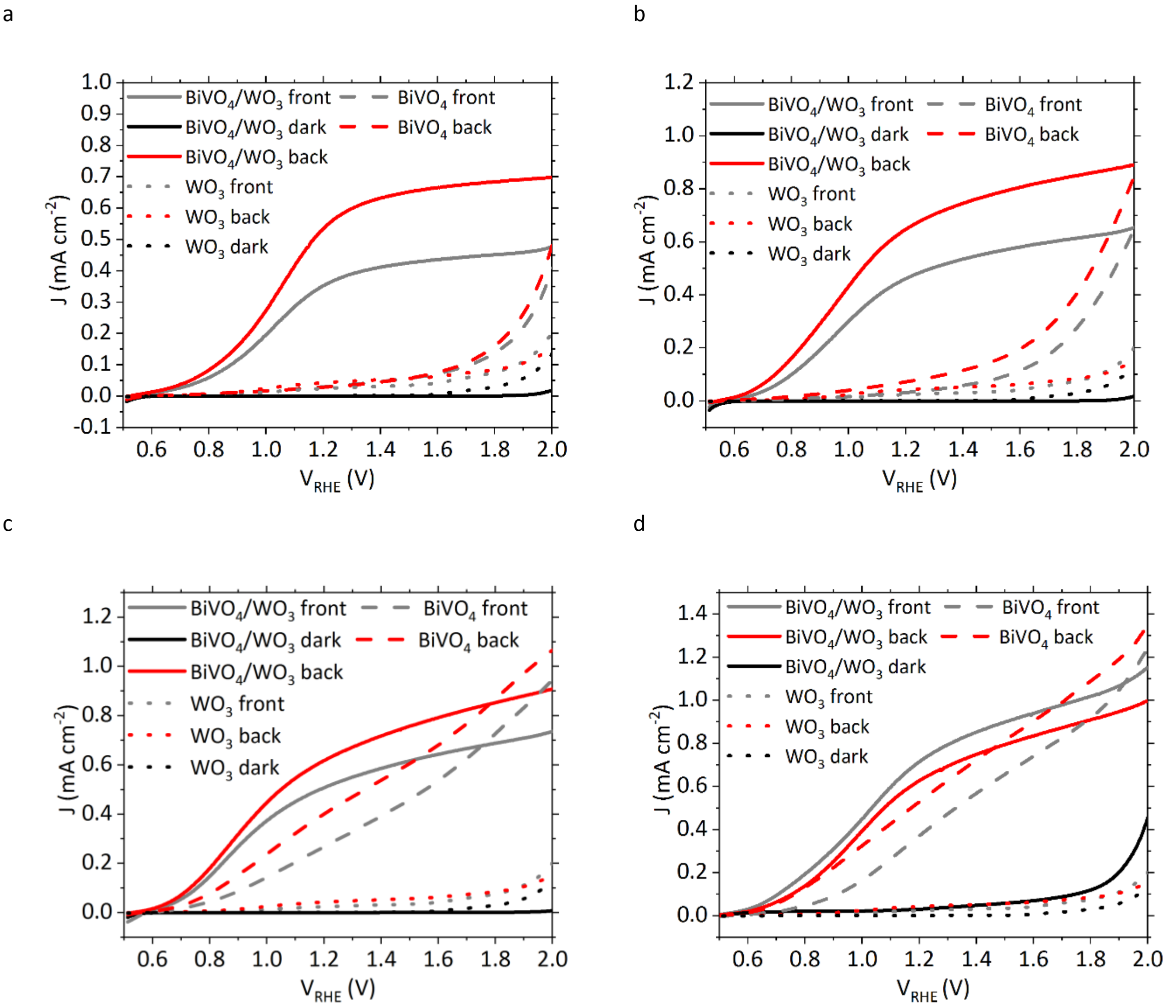

Fig. S5 JV curves of $WO_3/BiVO_4$ (solid line) with varying thicknesses of $BiVO_4$ (dashed lines) for thicknesses: (a) 75 nm, (b) 125 nm, (c) 175 nm and (d) 350 nm. The measurements were carried out in pH 7, 0.1 M phosphate buffer, under simulated 1 sun illumination (Xe lamp). The photoelectrochemical response of the WO_3 film is included for reference (dotted line).

IPCE and APCE. Incident photon to current efficiency (IPCE) is determined using the following equation:

$$IPCE = -\frac{J_{ph} \times 1239.8}{P_\lambda \times \lambda}$$

(Equation S1)

where J_{ph} (mA cm^{-2}) is the photocurrent density, 1239.8 (eV nm) is the Planck's constant multiplied by the speed of light, P_λ is the power intensity of the monochromatic light at a given wavelength, λ(nm).

To calculate the absorbed photon to current efficiency (APCE), firstly the photon flux of the simulated 1 sun illumination source (in figure S4) was determined below 500 nm, the absorption onset of the films.

$$Total\ no.of\ photons = \int_{350\ nm}^{500\ nm} Flux(\lambda) d\lambda$$

(Equation S2)

The absorptance of the films were also determined from the UV-vis data in the region: 350-500 nm using the equation:

$$Absorptance = 1 - 10^{-A}$$

(Equation S3)

Where A is the absorbance of the film.

The number of photons absorbed was then given by:

$$Number\ of\ photons\ absorbed = \int_{350\ nm}^{500\ nm} (Total\ no.of\ photons\ \times Absorptance) d\lambda$$

(Equation S4)

From the photocurrents obtained from the films at 1.23 V_{RHE}, the APCE was determined using:

$$\frac{J\ (A\ cm^{-2})/1.602 \times 10^{-19} C}{Number\ of\ photons\ absorbed\ (cm^{-2} s^{-1})}$$

Where 1.602 x 10^{-19} C is the elementary charge.

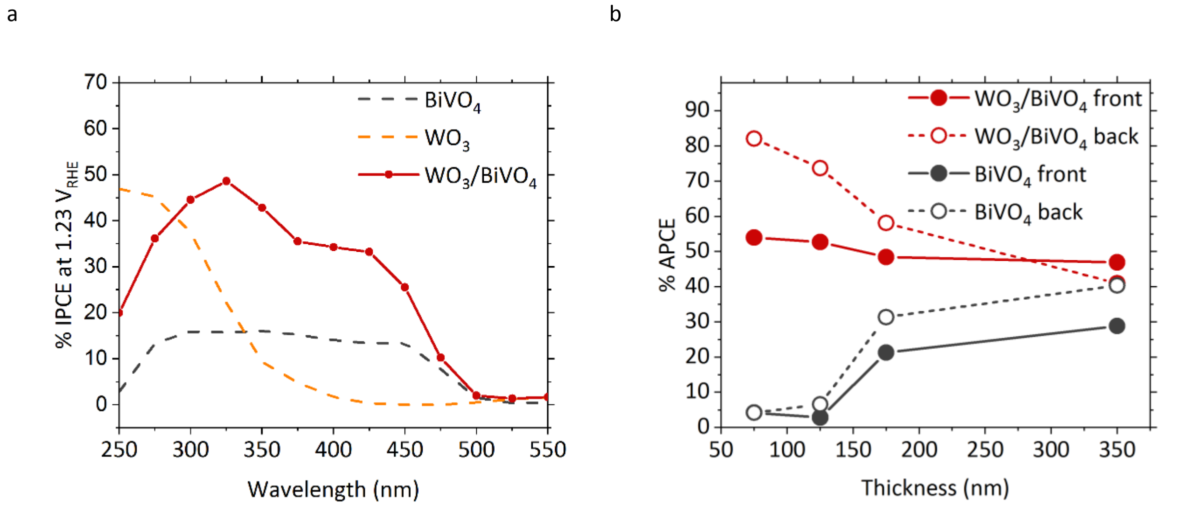

Fig. S6 IPCE under monochromatic light and APCE under simulated 1 sun, of films at 1.23 V_{RHE} (a) IPCE of $WO_3/BiVO_4$ heterojunction (red), illuminated from the front, with a $BiVO_4$ thickness of 350 nm. The IPCE of bare $BiVO_4$ (dark grey, dashed) and WO_3 (orange, dashed) are included for reference. (b) The APCE of $BiVO_4$ (dark grey) and $WO_3/BiVO_4$ (red) films with varying $BiVO_4$ thickness illuminated from the front (solid circles) and back (open circles).

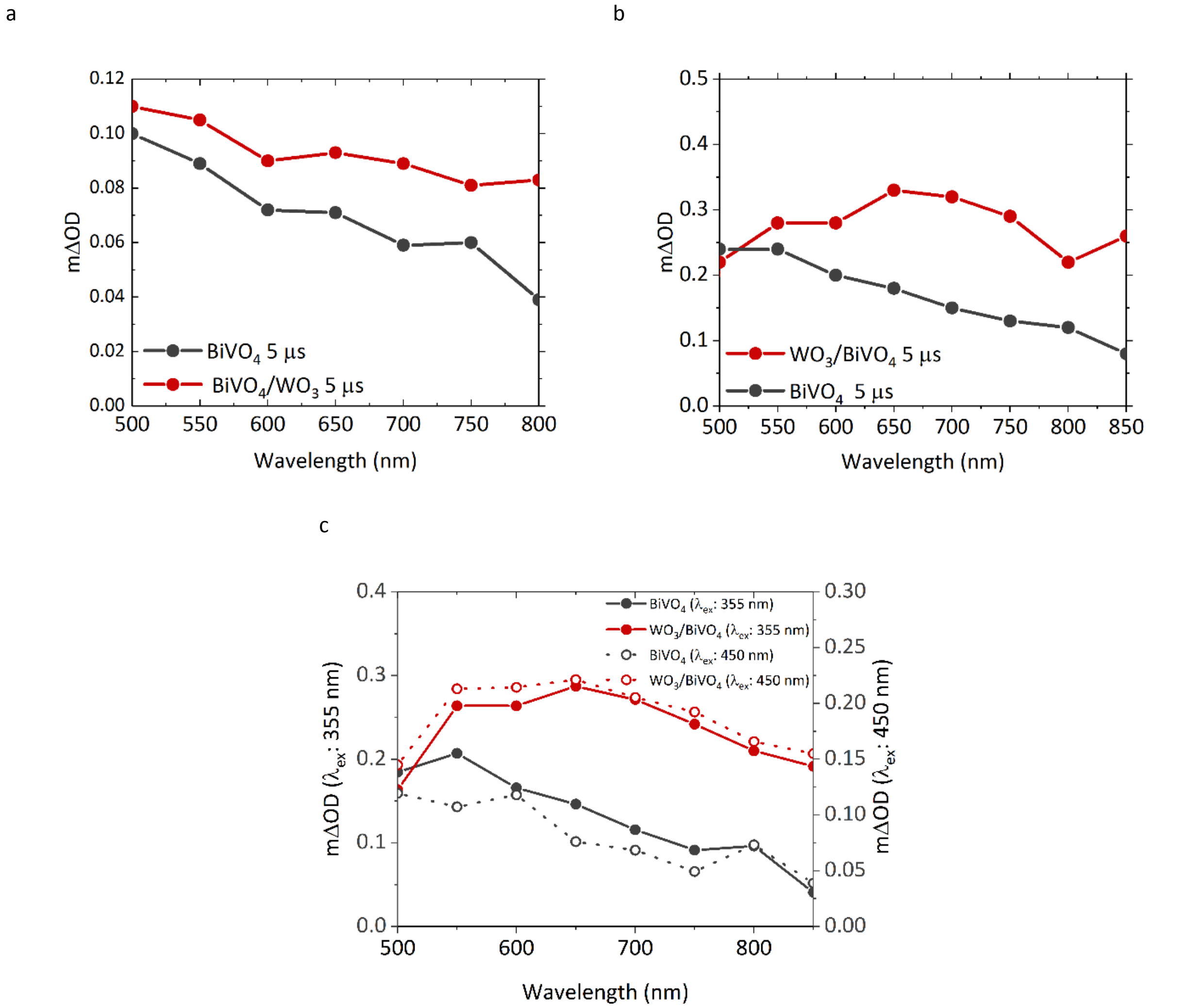

Fig. S7 Transient absorption spectra of $BiVO_4$ and $WO_3/BiVO_4$, front illumination, in 0.1 M phosphate buffer (pH 7); pump fluence: 500 μJ/cm^2 (355 nm) and 400 μJ/cm^2 (450 nm). Pump fluence was adjusted for normalised photon absorption by the sample. (a) $BiVO_4$ (grey) and WO_3/ $BiVO_4$ (red) at 5 μs, under no applied bias, excitation wavelength: 355 nm (b) $BiVO_4$ (grey) and WO_3/ $BiVO_4$ (red) at 5 μs, under 1.23 V_{RHE} applied bias, excitation wavelength: 355 nm. (c) Comparison of transient absorption spectra at 10 μs for $BiVO_4$ and $WO_3/BiVO_4$ when excited with 355 nm and 450 nm (exciting solely $BiVO_4$).

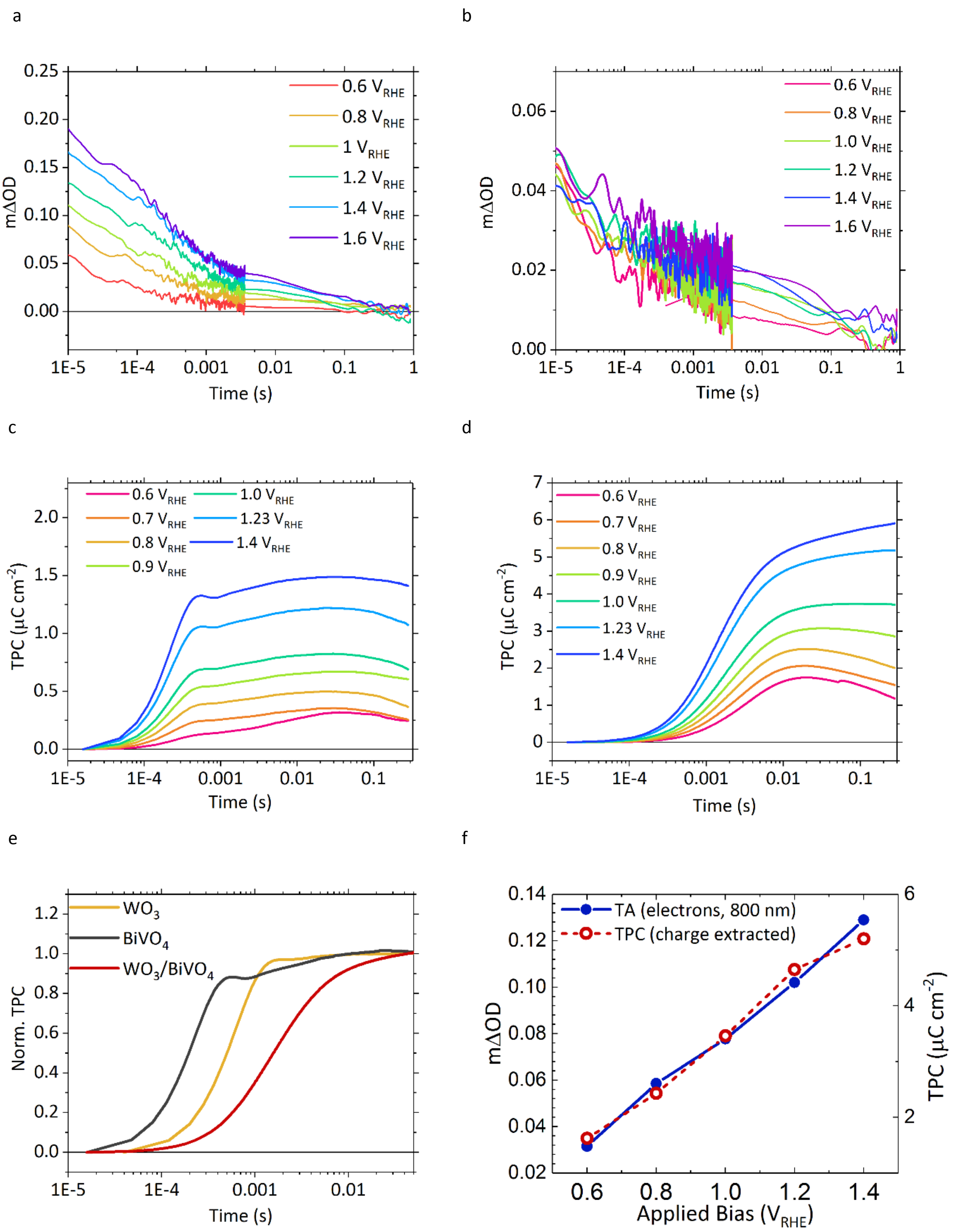

Fig. S8 TA kinetics and TPC of $BiVO_4$ and $WO_3/BiVO_4$ under front illumination, excited at 355 nm, with varying applied anodic bias in 0.1 M phosphate buffer; pump fluence: 500 μJ/cm^2. TA kinetic trace at 800 nm of (a) $WO_3/BiVO_4$, probing electrons in WO_3, and (b) $BiVO_4$ (absorption tail of broad h^+ absorption). TPC of (c) $BiVO_4$ and (d) $WO_3/BiVO_4$. (e) Normalised TPC at 0.01 s comparing electron extraction times of the heterojunction with that of the bare materials. (f) TA amplitude at 800 nm probing electrons in the $WO_3/BiVO_4$ system, at 50 μs (before the onset of charge extraction) (blue) compared with the total charge extracted from the $WO_3/BiVO_4$ photoanode (red).

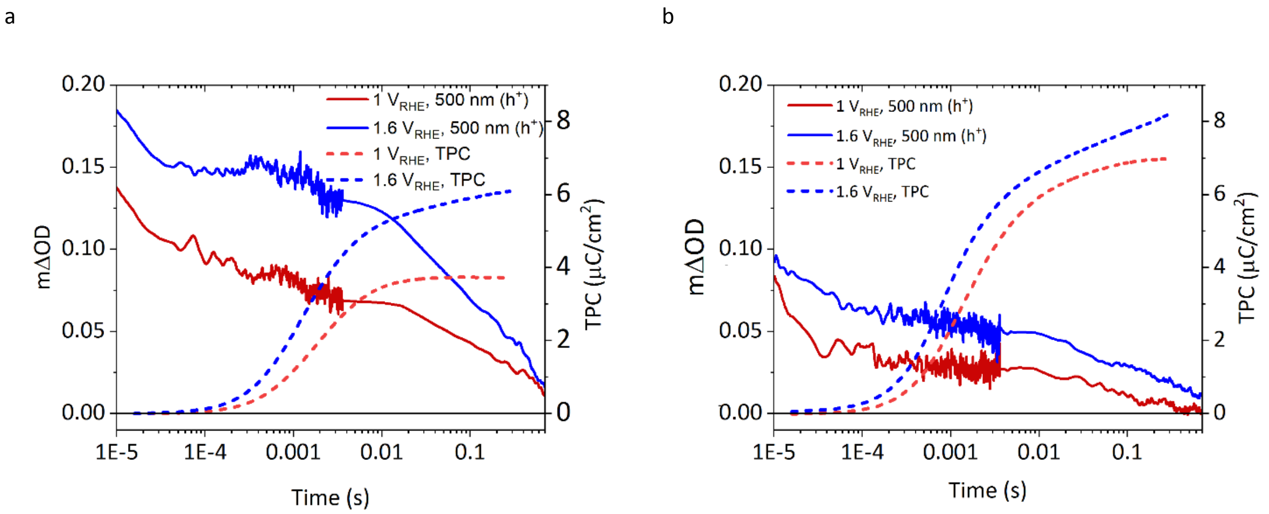

Fig. S9 Comparison of TA kinetics of holes accumulated in $BiVO_4$ (solid) against the integrated transient photocurrent, monitoring charge extraction from the $WO_3/BiVO_4$ heterojunction photoanode (dash), under applied potential of 1 V_{RHE} (red) and 1.6 V_{RHE} (blue) for (a) water oxidation and (b) sulphite oxidation. Water oxidation exhibits greater bias dependence in hole dynamics and greater hole accumulation, due to the slower kinetics of water oxidation, also reflected in the charge extracted from the film. Whereas for sulphite oxidation, the bias dependence is less pronounced, also shown in the charge extraction, due to sulphite oxidation being more facile in comparison to water oxidation. Less holes accumulate at the surface due to a faster reaction of holes to oxidise sulphite. The films were illuminated from the front ($BiVO_4$ side), excited at 355 nm, pump fluence: 500 μJ/cm^2, at 500 nm probing holes in $BiVO_4$, with and without hole scavenger. Hole scavenger used: 0.1 M Na_2SO_3.

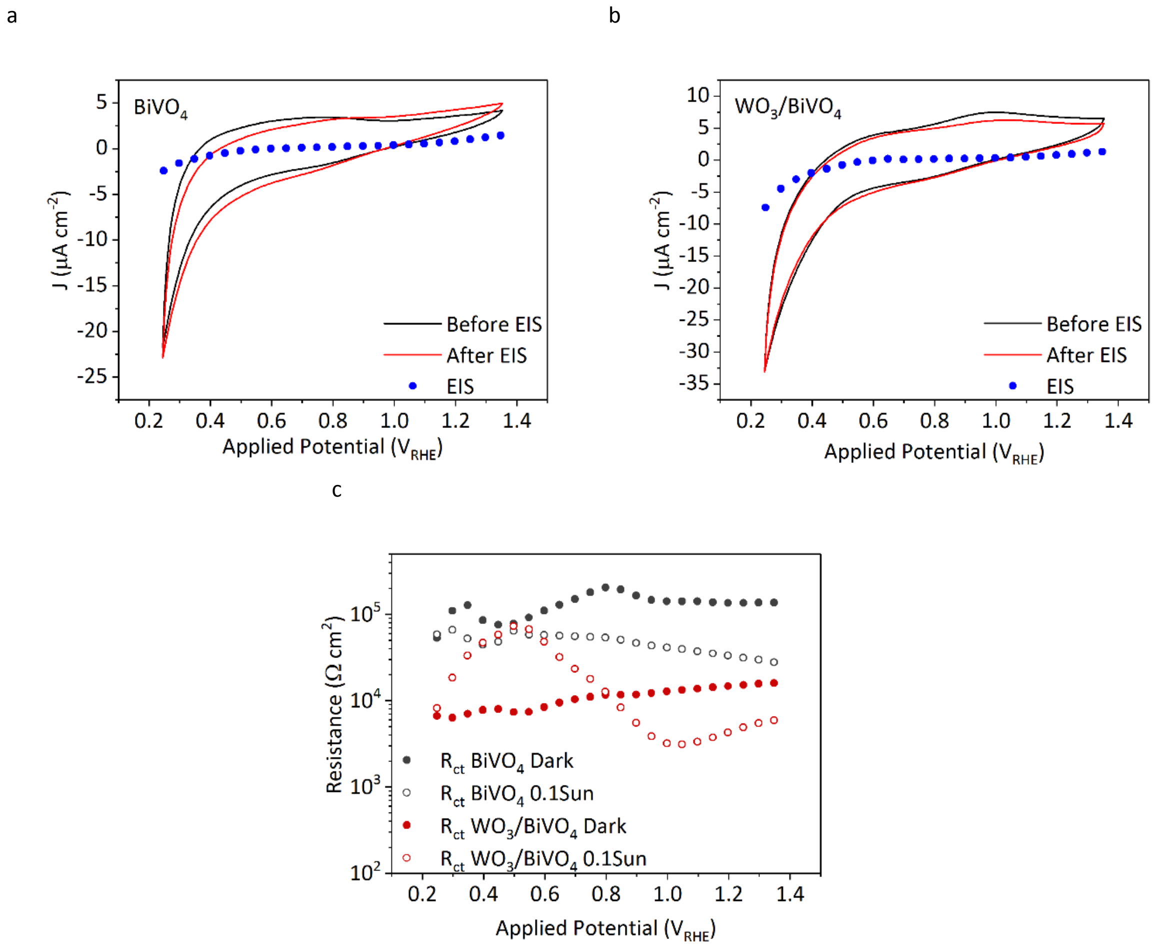

Fig. S10 Impedance analysis of $BiVO_4$ and $WO_3/BiVO_4$ photoanodes in 0.1M phosphate buffer. JV curves from cyclic voltammetry (black and red solid lines) and from impedance (blue dots) of (a) $BiVO_4$ and (b) $WO_3/BiVO_4$ in the dark. The good correspondence of both cyclic voltammetry and impedance JV curves indicates good stability of the films during the impedance measurements (c) Resistance of the photoanodes in the dark and under illumination (10% sun) showing the dependence of film resistance on applied potential. The voltage-dependent resistance for the $WO_3/BiVO_4$ sample reflects the slope of the steady-state j-V curve, since $R = \left(\frac{dj}{dV}\right)^{-1}$

Fitting of transient absorption kinetics as a function of applied potential

The kinetic traces were fitted using a model reported previously.[1]
The following equation was used to model the kinetic data:

$$\Delta OD = \alpha t^{-b} + A_{TAS2}\, e^{\frac{-t}{\tau(TAS2)}} + c$$

(Equation S5)

The parameters for $BiVO_4$:

Potential (V_{RHE})	Power law			Single exponential function	
	a	b	c*	A_{TAS2}	$\tau_{(TAS2)}$ (s)
**0.6**	3.47 x 10^{-6}	-0.27	4.60 x 10^{-6}	-	-
**1.2**	7.57 x 10^{-7}	-0.41	4.60 x 10^{-6}	2.05 x 10^{-5}	0.18
**1.4**	3.85 x 10^{-6}	-0.24	4.60 x 10^{-6}	2.70 x 10^{-5}	0.15

*the parameter c was used as an additional parameter for zeroing of kinetic traces

The parameters for $WO_3/BiVO_4$:

Potential (V_{RHE})	Power law			Single exponential function	
	a	b	c	A_{TAS2}	$\tau_{(TAS2)}$ (s)
**0.6**	4.75 x 10^{-6}	-0.22	-	-	-
**1.2**	3.53 x 10^{-7}	-0.26	-	2.05 x 10^{-5}	0.29
**1.4**	4.38 x 10^{-6}	-0.25	-	2.70 x 10^{-5}	0.22

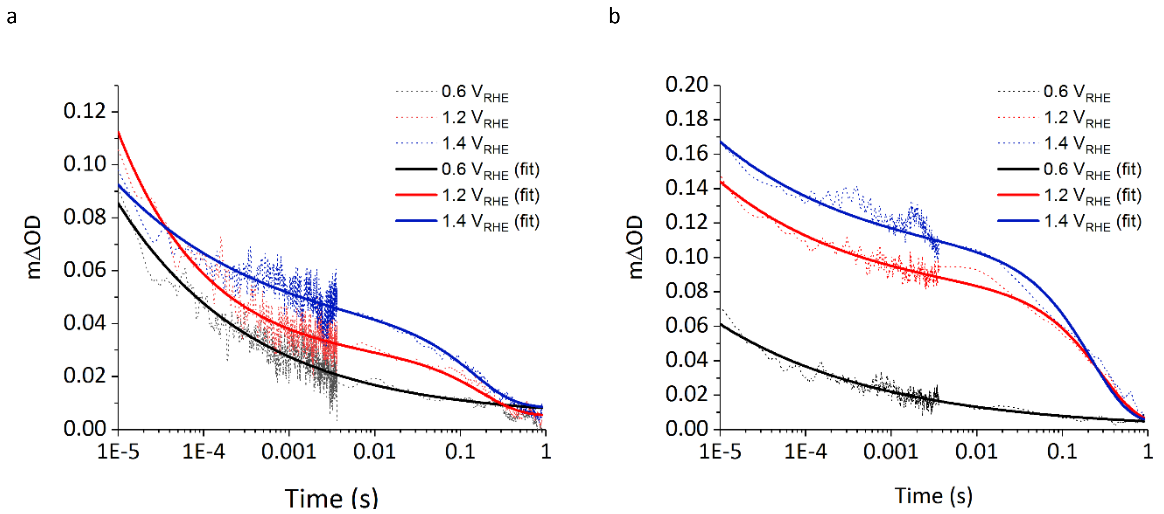

Fig. S11 Bias dependent kinetic traces at 500 nm probing hole dynamics and the corresponding fittings, measured at 0.6 V_{RHE} (black), 1.2 V_{RHE} (red) and 1.4 V_{RHE} (blue) applied potential; recorded in potassium phosphate buffer (0.1 M), excited at 355 nm at pump fluence of 500 μJ/cm^2. The traces were fitted using a combination of a power law and single exponential functions with parameters noted in Table S1. (a) $BiVO_4$ and (b) $WO_3/BiVO_4$.

From the fittings, the single exponential time constants represents the dynamics of holes accumulated within the space charge layer of the film. Comparison of these time constants between the $BiVO_4$ and the $WO_3/BiVO_4$ films highlights that the time constant for the composite is higher than that of $BiVO_4$. From previous studies, this slow component has been assigned to competing water oxidation and back electron/hole recombination processes and therefore the parameter is found to be applied-bias dependent. Being consistent with previous studies and the TPC data presented in this work, we observe that this back electron/hole recombination is significantly suppressed in the composite compared to bare $BiVO_4$.[1]

References

1 Y. Ma, S. R. Pendlebury, A. Reynal, F. Le Formal and J. R. Durrant, *Chem. Sci.*, 2014, **5**, 2964–2973.
